# The Socio-Environmental Impact in the Adjacent Settlements of a Former Foundry

**DOI:** 10.3390/ijerph22050692

**Published:** 2025-04-27

**Authors:** Griselda Vázquez-Quintero, Daniel Lira-Hernández, César Damián Pérez-Olmos, María Cecilia Valles-Aragón, Leonor Cortes-Palacios, César Guillermo García-González, Ireyli Zuluamy Iracheta-Lara, Myrna Concepción Nevárez-Rodríguez, Gilberto Sandino Aquino-de los Ríos

**Affiliations:** 1 Faculty of Agricultural Sciences, Autonomous University of Chihuahua, Pascual Orozco Avenue s/n, Campus I, Chihuahua 31200, Chihuahua, Mexico; gquintero@uach.mx (G.V.-Q.);; 2 Faculty of Zootechnic and Ecology, Autonomous University of Chihuahua, Periférico Francisco R. Almada Km 1, Chihuahua 31453, Chihuahua, Mexico; lcortes@uach.mx (L.C.-P.);

**Keywords:** metals and metalloids, mining waste, social perception

## Abstract

Mining has caused major pollution, especially in poorly regulated areas. The former Ávalos Foundry in Chihuahua, Mexico left toxic contamination after its closure in 1997, affecting the nearby settlements. This study examines the socio-environmental impact on residents adjacent to the site. A total of 5773 dwellings were considered, with 4634 inhabited by 14,187 persons. A survey to 465 residents assessed sociodemographic aspects, environmental perceptions, and disposition to community participation. Tap water samples from 70 homes were analyzed for metals and compared to Mexican, American and European regulatory standards. Water pollutant dispersion was modeled using ArcGIS interpolation. Residents face economic, social, environmental, and health issues from ongoing contamination. Several suffer respiratory and skin diseases linked to excessive dust from the proximity to mining waste and unpaved streets. While the majority consider their lives comfortable or very comfortable, many would not have moved there if aware of the risks before moving. Despite concerns, most residents are reluctant to engage in community efforts to address the pollution. Tap water tests revealed levels above the regulatory standards of arsenic, copper, chromium, iron, manganese, and nickel, posing serious health risks. This study calls for immediate action, including awareness and health campaigns, environmental remediation, and intersectoral collaboration to secure funding for long-term solutions.

## 1. Introduction

Mining has long been an important source of economic resources, but it has left a legacy of pollution that has had detrimental consequences for human health and the environment. Despite the significant revenue it has generated, especially during metal boom periods, workers’ movement to mining areas has not always been accompanied by adequate planning to mitigate the negative impacts. In addition, the use of outdated mining methods and the lack of compliance with environmental, mining, and health regulations have aggravated the problems associated with this activity [1].

One of the most significant risks associated with mining activity is the improper management of waste, which contains highly toxic components such as arsenic, cadmium, copper, lead, mercury, chromium, selenium, and zinc, among others [2]. The adverse effects of exposure to toxic metals are especially concerning in vulnerable populations, such as children and pregnant women. From prenatal development to adulthood and aging, exposure to those pollutants can cause a wide range of health problems, ranging from alterations in physical and intellectual development to reproductive effects and the increased risk of certain types of cancer [3].

The Ávalos Foundry plant, located in the southeast of Chihuahua City, Mexico began operations in 1908. This site was one of the major industries in the region, processing mineral concentrates through pyrometallurgical processes [4].

Following the full stop of operations in 1997, the zone went from being an industrial center to becoming a focus of pollution. With more than 324 hectares affected, it represents the biggest environmental liability in Latin America [4]. Therefore, the Government of the State of Chihuahua acquired through a trust the entire area corresponding to the former foundry Ávalos with the intention of remediating the area [5]. Due to this acquisition, the zone experienced a change in its urban use, going from being an industrial-oriented to a service-oriented area, with the construction of bus terminals, supermarkets, government offices, and other public facilities [6]. In 2003, a real estate company promoted the change in land use of the adjacent area to the mining tailings, leading to the approval of the housing development of the Rinconada Los Nogales neighborhood during the 2004–2007 administration [7]. Hence, unfortunately the contamination left has continued affecting the health and well-being of the surrounding residents [6]; lead particles and other wastes containing heavy metals in the soil have significantly affected nearby human settlements [4], as evidenced by the numerous complaints and lawsuits from nearby residents [6].

There are various official and unofficial documents that contextualize the history and problems of Ávalos; however, there is not much “public” information to support the research that demonstrates the environmental problems in the site. Ornelas-Hicks et al. (2007) determined the lead concentration in the soil and blood from mothers and preschool children, which indicated a serious problem was evident, especially for children [8]. Subsequently, there have been several studies characterizing some metals and metalloids, particularly Pb, in mining tailings and residential soil, all evidencing that there is a contamination problem in the area [8,9,10,11,12]. However, none of this research contextualizes the problem experienced by the inhabitants, nor the population’s opinion on their quality of life.

Despite government efforts to address the problem, including remediation plans announced in 2016 and 2017, those have not achieved an effective solution to mitigate the pollution impacts [4]. The lack of action has generated growing concern among the affected people and has led the National Human Rights Commission (CNDH) to intervene, ordering urgent measures to protect the health and rights of the inhabitants in the affected neighborhoods [6]. Therefore, in 2022 it was of interest to know the population’s perspective on the condition of their quality of life. To this effect, a first instrument was developed and applied in the Rinconada Los Nogales neighborhood [13]. However, it was determined that although the CNDH primarily observes the situation of the closest settlement, there is more population affected by the environmental liability.

The lack of effective solutions has led to a state of abandonment of both industrial facilities and local activities, leaving a legacy of pollution and health problems that persist to this day [4]. Although, the former foundry in Chihuahua represents a significant source of concern, there is a notable lack of attention to the socio-environmental conditions of the adjacent human settlements. This research seeks to address this gap by analyzing the current context in these households.

## 2. Materials and Methods

### 2.1. Study Area

**Figure 1:**
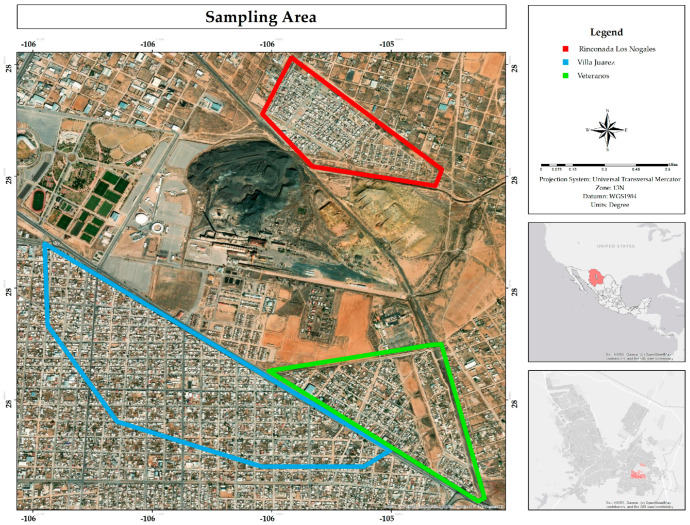
Sampling areas.

The study area is located near the former Ávalos smelter plant in the southeastern part of Chihuahua City, Mexico. The residential areas considered in this research were Villa Juarez (blue polygon), Veteranos (green polygon) and Rinconada Los Nogales (red polygon) (Figure 1).

### 2.2. Demographic and Housing Analysis in the Study Area

According to data by the National Housing Institute (INVI) there are a total of 5773 housing units in the study area, of which 4634 are currently inhabited. The total population residing in this area is 14,187 individuals. Of this population, those considered eligible to participate in the evaluation instrument were individuals over 14 years of age, totaling 11,252 persons (Table 1 and Table 2) [14].

Based on the secondary zoning of the study area, as outlined in the Urban Development Plan of the Municipality of Chihuahua 2040 (PDU) in its seventh update [5], Rinconada Los Nogales has a housing density classification of H45, meaning a permitted housing density of 45 dwellings per hectare. In contrast, the surrounding area, which includes even farms, has a mixed-suburban land-use classification, subdivided into MS and MS2, allowing for a maximum density of H45 and H35 (45 and 35 dwellings per hectare), respectively.

Meanwhile, the other neighborhoods in the study area (Villa Juarez and Veteranos) have a commercial land use along some main avenues and share the housing density classification of H35.

### 2.3. Population Perspective

#### 2.3.1. Evaluation Instrument

An assessment instrument was developed in the software ArcGis Survey 123 (ESRI, Redlands, CA, USA), comprising 28 questions structured across different measurement scales: nominal, ordinal, and interval. The objective of this instrument was to analyze key aspects of the people residing in the area next to the former foundry, categorized into the following dimensions:

- Sociodemographic characteristics: included variables such as sex, age, household size, presence of more vulnerable individuals (under 18 and older than 60), and residence time in the area.
- Perception of the residential environment: evaluated factors influencing settlement decisions, perceived comfort levels, awareness of environmental and health risks, and reported symptoms or health conditions associated with proximity to the mining waste.
- Expectations from public administration and commitment: Identified priority actions from the resident’s perspective and highlighted infrastructure and service deficiencies. In addition, it analyzed the resident involvement level in activities related to environmental conservation and local improvement.

#### 2.3.2. Sample Size

The sample size was determined with a 95% confidence level and a 5% error margin. The minimum number of surveys required to statistically represent the entire population older than 14 years old (11,252 habitants, Table 2) was calculated to be 372. The estimated values were calculated using Equation (1).

(1) $$n=\frac{N\sigma^{2}Z^{2}}{Ne^{2}+Z^{2}\sigma^{2}}$$

where:

*n* = sample size;

*N* = population size (11,252 population older than 14 years old;

*σ* = standard deviation (0.05 recommended to ensure the enough sample size);

*Z* = confidence level constant (1.96 for a 95% confidence level);

*e* = margin of error (5% recommended as a standard value).

To ensure an adequate sample, 500 sampling points were planned, of which 465 surveys were successfully conducted and distributed proportionally according to the number of housing units per neighborhood in the study area, as follows: Villa Juarez (280 surveys), Rinconada Los Nogales (98 surveys), and Veteranos (87 surveys).

#### 2.3.3. Data Processing and Analysis

Data from ArcGis Survey 123 were downloaded in Excel format; these were reviewed and grouped, considering some answers that were very different from the rest as “other” and excluding the unanswered questions. Percentages were calculated, graphics were made, and attributed tables were constructed for mapping answers in the ArcGis 10.8 software (ESRI, Redlands, CA, USA).

### 2.4. Metals in Water

#### 2.4.1. Sampling

Water samples were collected in 1 L plastic bottles from 68 household taps in the residences from the study area, supplied by the municipal network, sourced from ground water distant from the study area. However, each household stores water in elevated tanks, which are vulnerable to airborne suspended particles, particularly if left uncovered or poorly maintained. A non-probabilistic convenience sampling method was used, depending on the willingness of residents to allow water collection from their homes.

#### 2.4.2. Sample Preparation and Analysis

The water samples underwent an acid digestion process using an Anton Paar Multiwave Go digester, following the EPA 3015A method, with 10 mL of HNO_3_ and 4 mL of HCl. Metal and metalloid concentrations were determined using an Inductively Coupled Plasma Optical Emission Spectrometer (ICP-OES) (Perkin Elmer OPTIM-8300, PerkinElmer, Inc., Waltham, MA, USA), with blanks and triplicate samples to ensure proper standard deviation and error calculation. The elements analyzed include arsenic (As), cadmium (Cd), copper (Cu), chromium (Cr), iron (Fe), manganese (Mn), nickel (Ni), lead (Pb), selenium (Se), beryllium (Be), cobalt (Co), titanium (Ti), thallium (TI), and zinc (Zn). The metal and metalloid concentrations were compared against the maximum permissible limits established by NOM-127-SSA1-2021 for water intended for human use and consumption [15], the United States Environmental Protection Agency in the National Primary Drinking Water Regulations [16], and Directive European Unión (EU) 2020/2184 on the quality of water intended for human consumption [17].

### 2.5. Data Analysis and Modeling

Data obtained were processed in Excel. The mean and standard deviation were calculated. These data were also visualized using the ArcGis 10.8 software (ESRI, Redlands, CA, USA) to model contaminant dispersion in sector maps. The GeoStatistical Analyst Wizard in ArcGis 10.8 software (ESRI, Redlands, CA, USA) extension was used, employing the Inverse Distance Weighted (IDW) interpolation method.

The IDW method estimates the concentration of unsampled sites using existing values from the research area, where observations closest to the site have a greater influence than those farther away, with influence decreasing as distance increases. The estimated values were calculated using Equation (2).

(2) $$Z\left(S_{0}\right)=\sum_{i=l}^{N}\lambda \;x\;Z(S_{i})$$

where:

*Z*(*S*_0_) = the value to be estimated in place *S*_0_;

*N* = the number of observations close to the place to be estimated;

*λ* = the weight assigned to each observation to be used, where weight decreases with distance;

*Z*(*S_i_*) = the observed value of place *S_i_*.

## 3. Results

### 3.1. Sociodemographic Characteristic

A total of 465 inhabitants were interviewed, of which 52% were women and 41% were men, while 5% did not specify their gender. The respondents were all over 14 years old; the age group with the highest number of responses was the 45 to 49 age range, representing 12% of the total, followed by the 55 to 59 age group with 11%, and the 60 to 64 age group, which provided 11% of the responses. Overall, adults above 45 years old constituted the predominant response groups, accumulating 34%.

**Figure 2:**
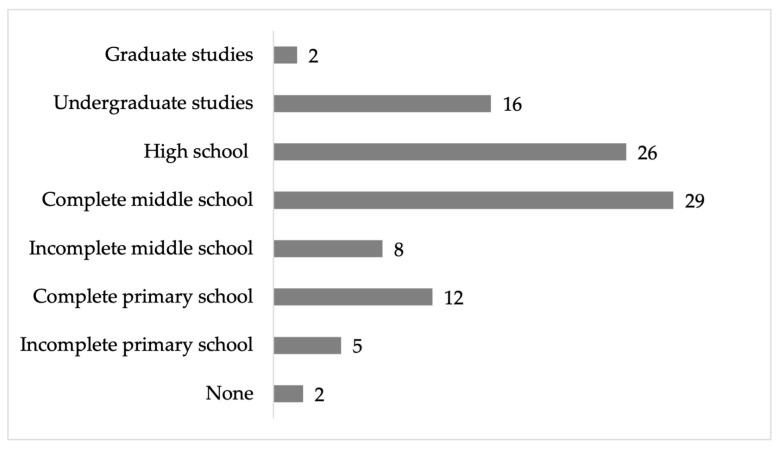
Percentage of the educational level of the respondents in the residential area surrounding the former Ávalos foundry.

**Figure 3:**
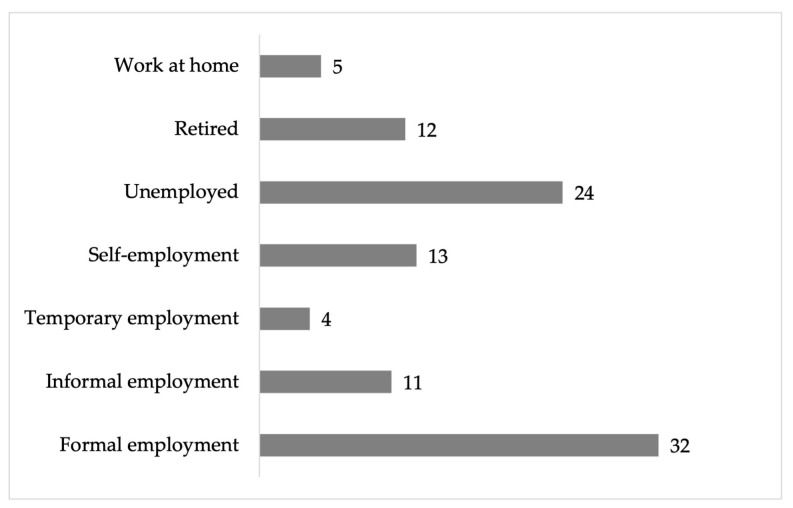
Percentage of the employment status of the respondents in the residential area surrounding the former Ávalos foundry.

Of the people surveyed, 29% had completed middle school, while 26% reached high school; only 16% finished undergraduate studies, and 2% had completed graduate studies (the maximum level that can achieved) (Figure 2). A total of 32% of the respondents have a formal job, which includes benefits such as vacations, social security, and a stable income [18]. On the other hand, 24% of respondents reported being unemployed (Figure 3).

**Figure 4:**
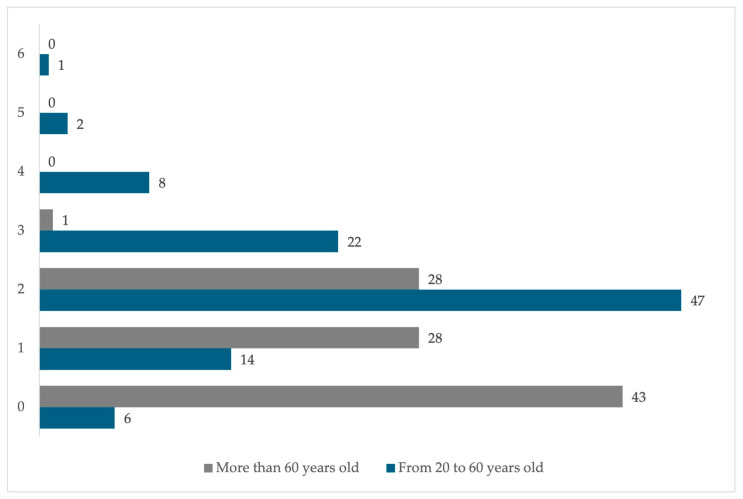
Percentage of adults aged 20 to 60 and 60 years and older per dwelling in the residential area surrounding the former Ávalos foundry.

**Figure 5:**
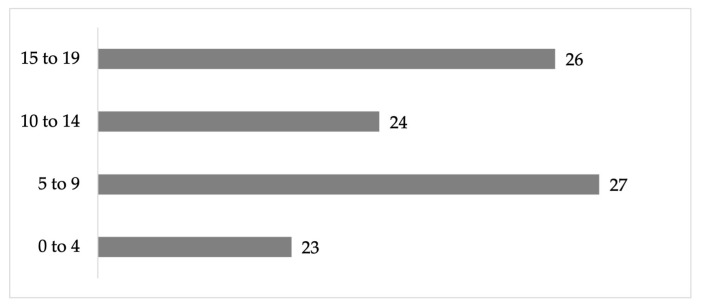
Percentage of children per dwelling in the residential area surrounding the former Ávalos foundry.

The dwellings are predominantly inhabited by two adults per dwelling from 20 to 60 years of age (47%), zero adults over 60 years of age per dwelling (43%), or dwellings with one or two adults aged 60 or older (with 28% in each category) (Figure 4). In addition, the dwellings are inhabited by children or young people in the 0 to 19 age group, with the dominant groups being 5 to 9 years (27%) and 15 to 19 (26%) (Figure 5).

**Figure 6:**
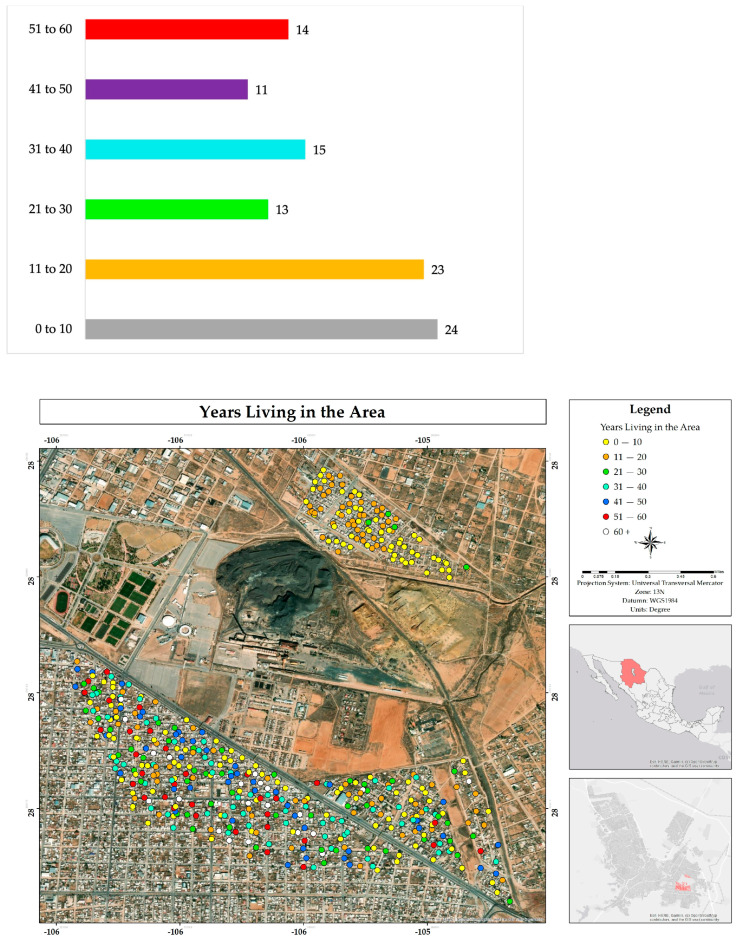
Percentage and spatial distribution of years living in the residential area surrounding the former Ávalos foundry.

The inhabitants of the study area have been living in the area for between 0 and 60 years or more. The most frequently mentioned time ranges were 0 to 10 years (24%) and 11 to 20 years (23%). This trend was observed more clearly in the Rinconada Los Nogales neighborhood, since it was established in 2005. In contrast, in the other two neighborhoods analyzed, a greater diversity of responses was obtained (Figure 6).

**Figure 7:**
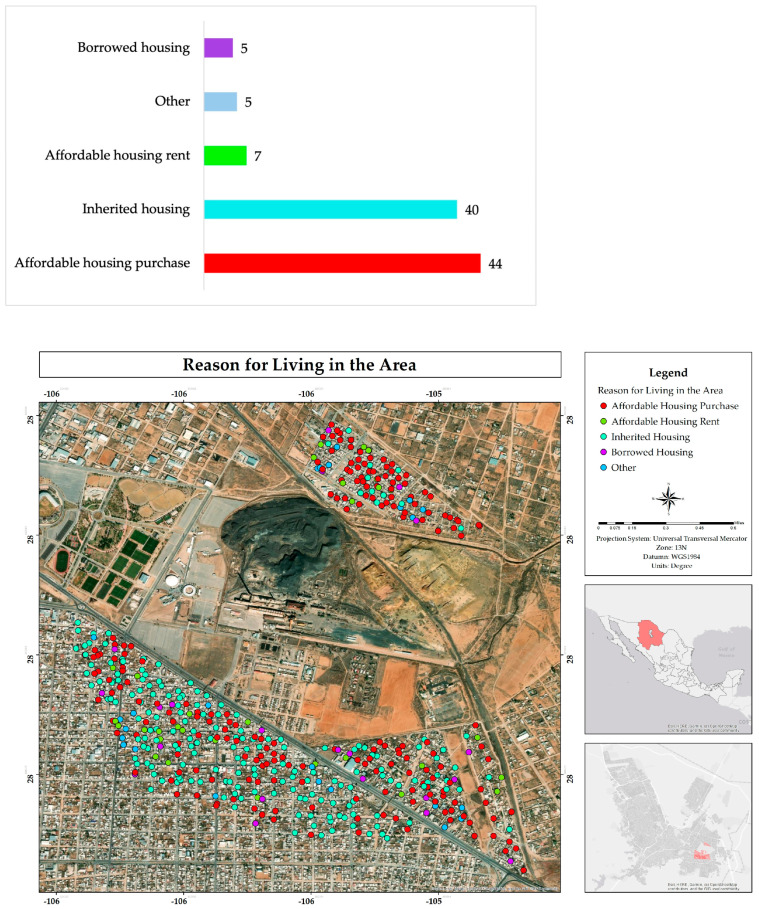
Percentage and spatial distribution of the reasons for residing in the residential area surrounding the former Ávalos foundry.

The main reasons indicated for choosing or remaining in this area were the affordable price of housing (44%) and the acquisition of housing through inheritance (40%). In the Rinconada Los Nogales neighborhood, the influence of the cost of housing predominates, while in the other two neighborhoods analyzed, both conditions have a significant weight (Figure 7).

### 3.2. Perception of the Residential Environment

**Figure 8:**
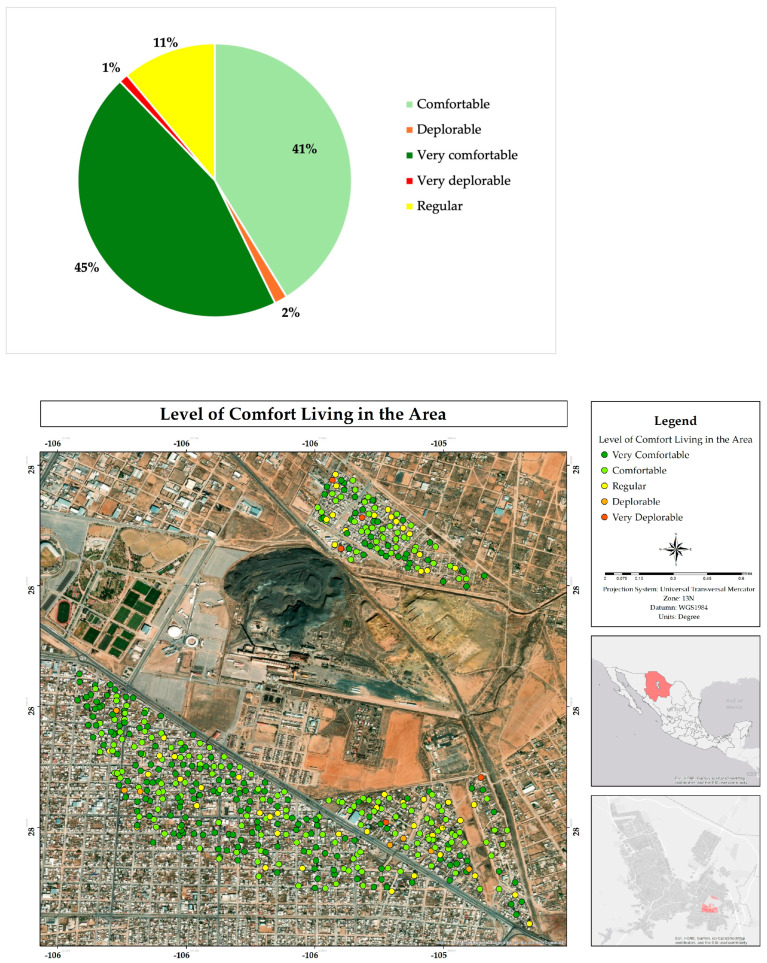
Percentage and spatial distribution of the comfort degree of living in the residential area adjacent to the former Ávalos foundry.

Analysis of residents’ perceptions of the comfort level in their surroundings revealed that 86% of residents consider that they live comfortably or even very comfortably (Figure 8). This high percentage suggests a strong association with the sense of belonging that residents have developed towards the place [19].

**Figure 9:**
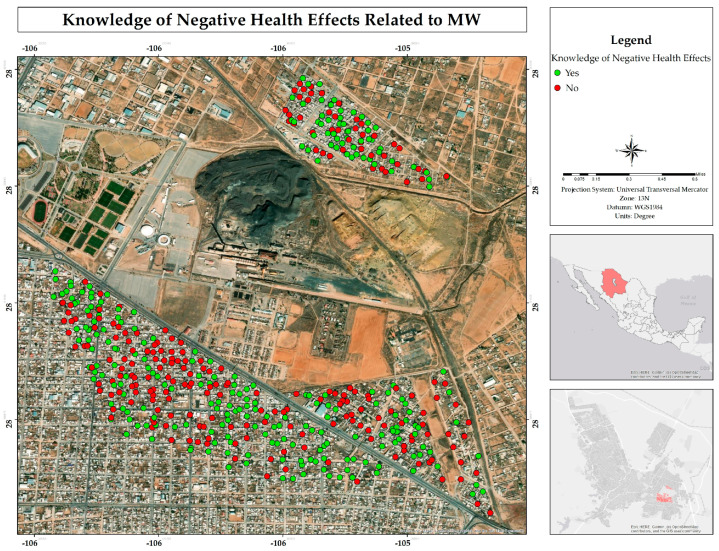
Spatial distribution of knowledge about the negative health effects in the residential area surrounding the former Ávalos foundry.

**Figure 10:**
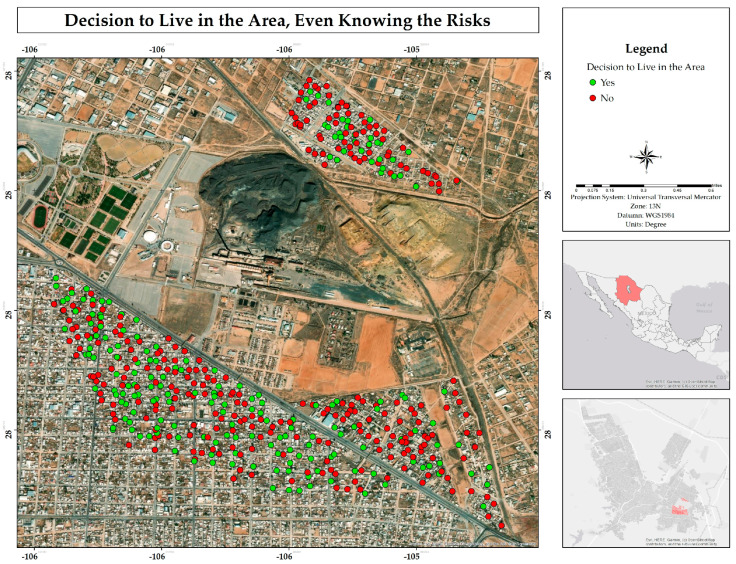
Spatial distribution of the decision to live in the area, even knowing the risks associated with living adjacent to the former Ávalos smelter.

Although 53% of residents claimed to be informed about the risks associated with living near mining waste (Figure 9), 59% indicated that had they had known about these risks in advance, they would have chosen not to settle in the area (Figure 10). This result represents a paradox, since those who claimed to be aware of the risks still decided to reside in the place, while the majority said that they would not have done so if they had known about the dangers from the beginning. Given that information, most people exposed to slowly or rapidly deteriorating environmental conditions do not migrate despite the availability of exit options [20].

**Figure 11:**
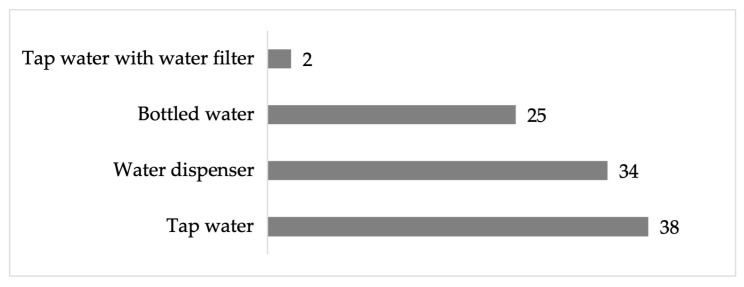
Percentage of water consumption sources in the residential area surrounding the former Ávalos foundry.

Respondents indicated that the source of water they use is direct tap water (38%) or a water dispenser (34%). Some consume bottled water (25%), and others mentioned that their source of supply was tap water with a filter to improve the quality of the water consumed (2%) (Figure 11).

**Figure 12:**
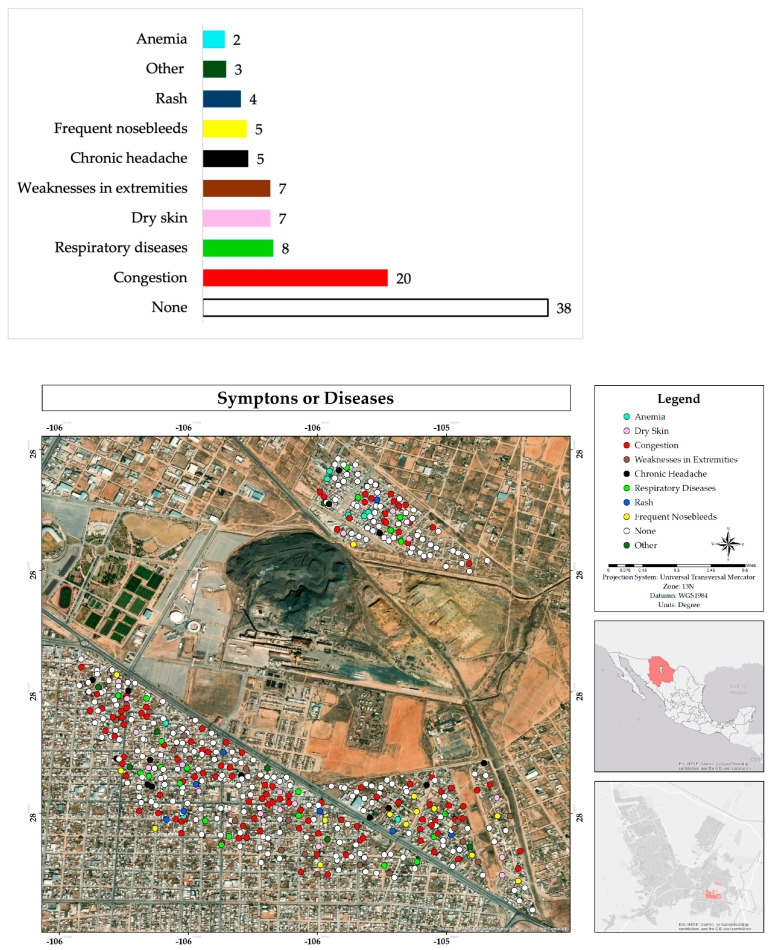
Percentage and spatial distribution of the symptoms or illnesses perception of respondents or family members who live in their homes in the residential area adjacent to the former Ávalos foundry.

Most of the inhabitants of the area indicated that neither they nor their family members living in the house suffer from symptoms of diseases (38%); however, 28% mentioned suffering from nasal congestion and respiratory diseases. There was also an incidence of responses of presenting dry skin (7%), extremities weakness (7%), chronic headache (5%), frequent nosebleeds (5%), and rash (4%). In addition, in the others section they mentioned hypertension, diabetes, and dizziness (Figure 12).

**Figure 13:**
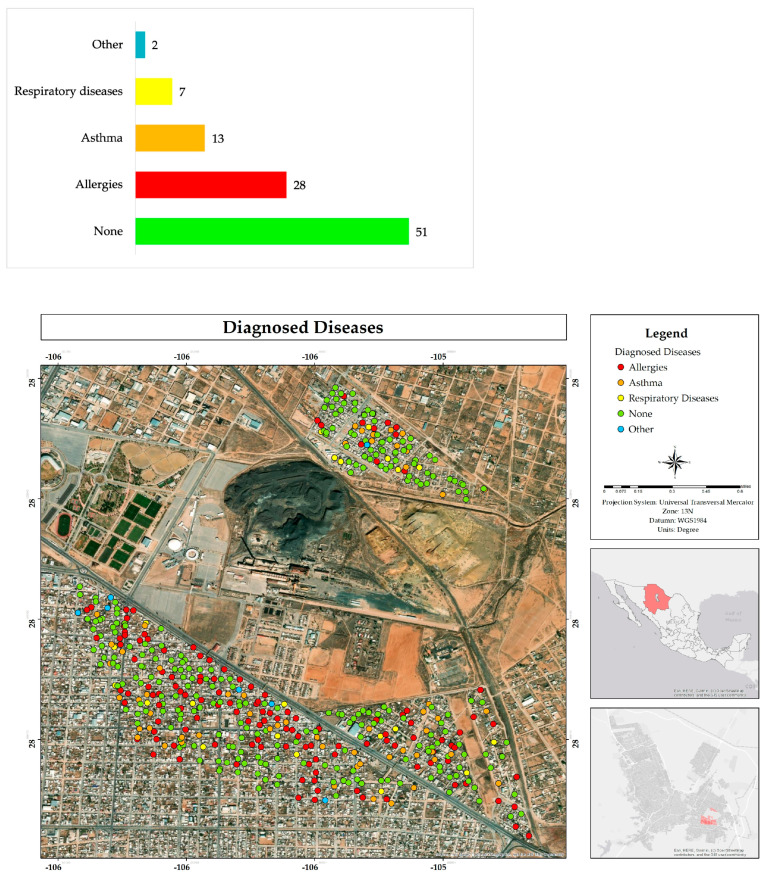
Percentage and spatial distribution of diseases diagnosed in respondents or family members residing in their homes in the residential area adjacent to the former Ávalos foundry.

The respondents indicated that they or family members living in their house have not been diagnosed with any disease (51%); they reported suffering allergies (28%), as well as asthma or other respiratory diseases (20%). In other diagnosed diseases they mentioned osteoporosis, cancer, high blood pressure, and diabetes (Figure 13).

**Figure 14:**
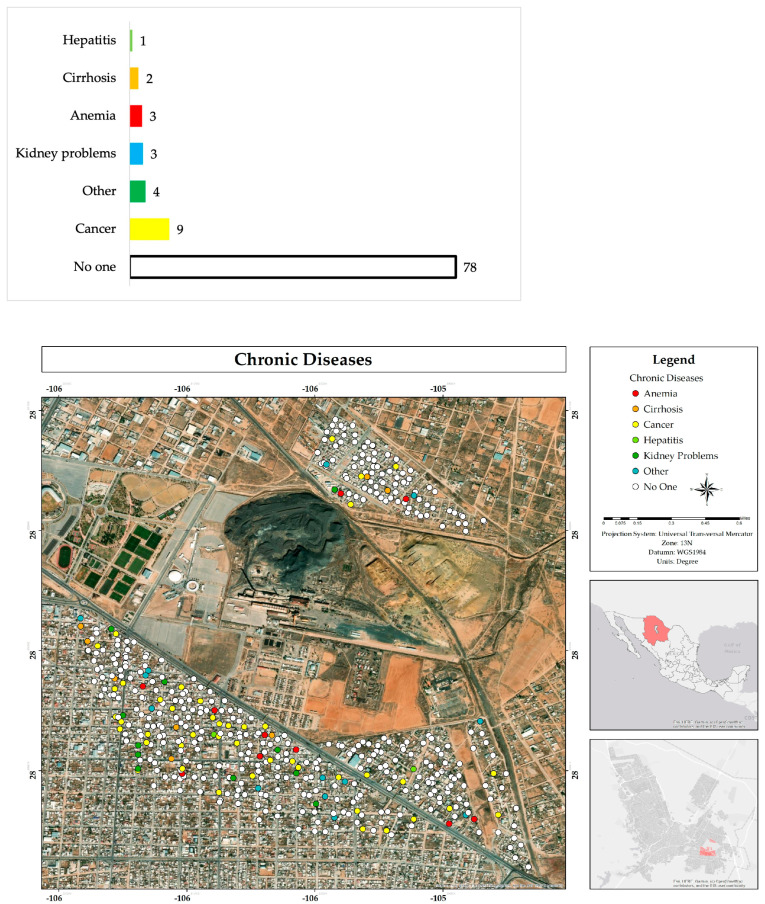
Percentage and spatial distribution of the knowledge about chronic diseases suffered by neighbors of the respondents in the residential area adjacent to the former Ávalos foundry.

**Figure 15:**
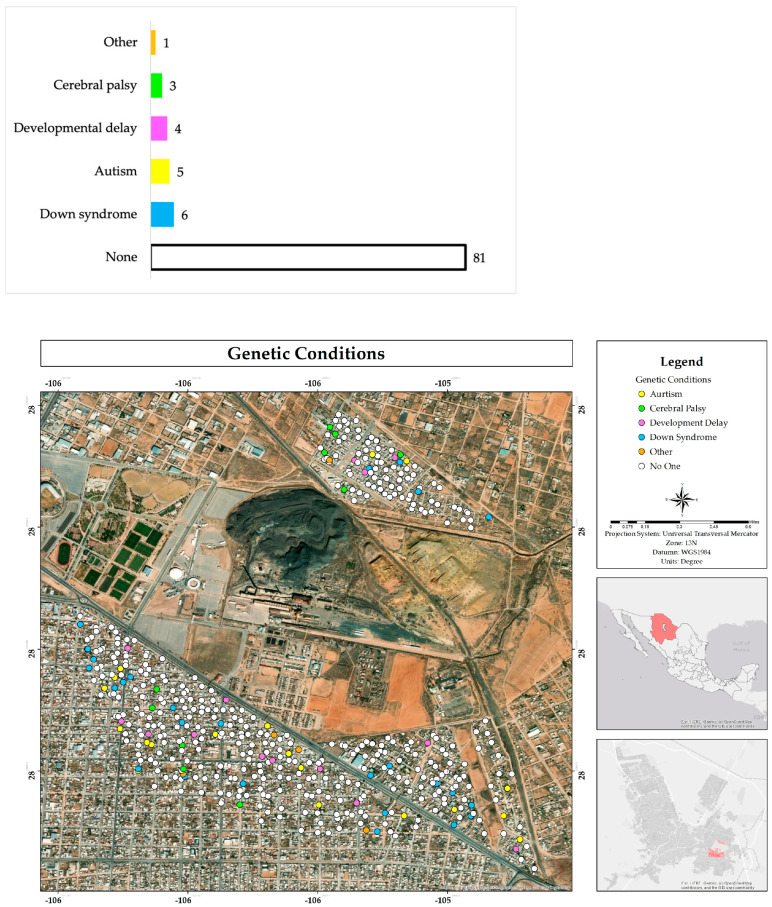
Percentage and spatial distribution of knowledge about genetic conditions suffered by neighbors of the respondents in the residential area adjacent to the former Ávalos foundry.

Most people do not know anyone on their street who suffers from any type of chronic disease (78%) or genetic problems from birth (81%). The most frequently mentioned disease was cancer (9%), as well as kidney problems (3%), anemia (3%), and cirrhosis (2%), among others. As for birth genetic conditions, the most frequent were Down syndrome (6%), autism (5%), developmental delay (4%), and cerebral palsy (3%) (Figure 14 and Figure 15).

**Figure 16:**
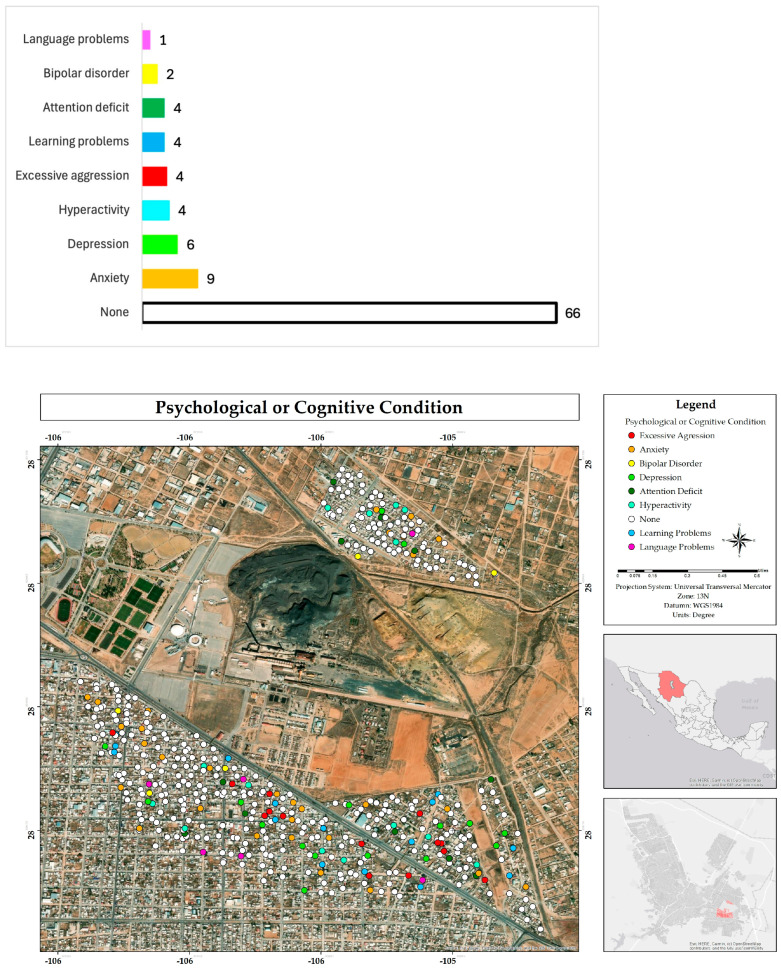
Percentage and spatial distribution of the perception of cognitive or psychological disorders of respondents in the residential area surrounding the former Ávalos foundry.

Likewise, most respondents indicated that neither they nor anyone in their family living in their home suffers from any psychological or cognitive condition (66%); the dominant conditions were anxiety (9%), followed by depression (6%), hyperactivity (4%), and excessive aggressiveness (4%) (Figure 16).

**Figure 17:**
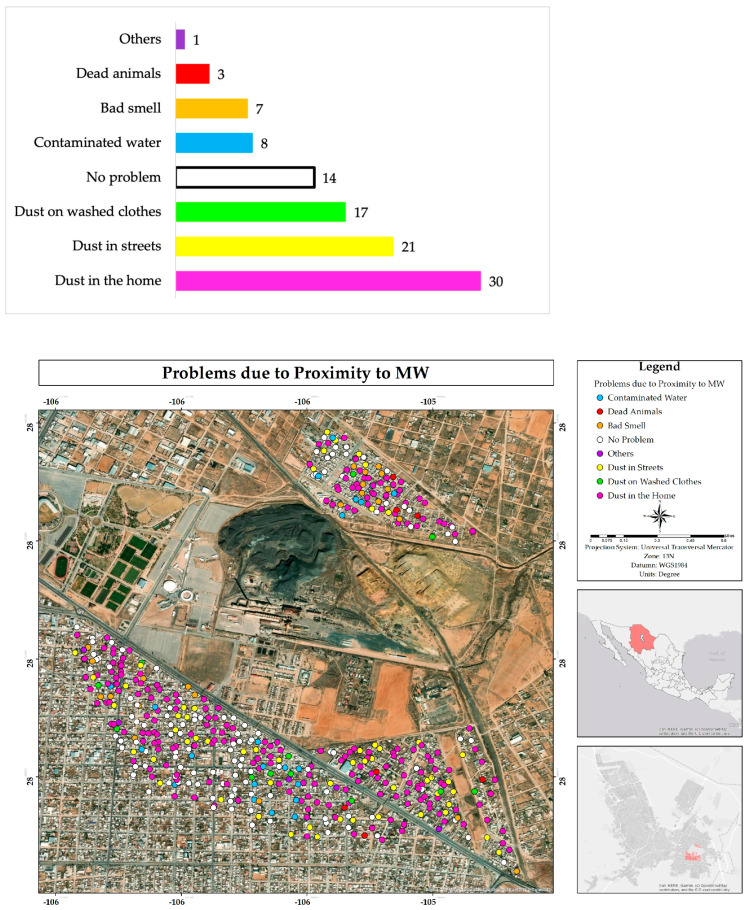
Percentage and spatial distribution of problems faced by respondents in the residential area surrounding the former Ávalos foundry.

The main problems identified by residents of the study area arising from proximity to mining waste were dust in homes (30%), in the streets (21%), and clothing (17%), in a total of 68% of the responses. Residents also mentioned contaminated water, bad smells, dead animals, lack of paving in the streets, water cuts, the presence of stray dogs, and the insecurity generated by the presence of immigrants (Figure 17).

**Figure 18:**
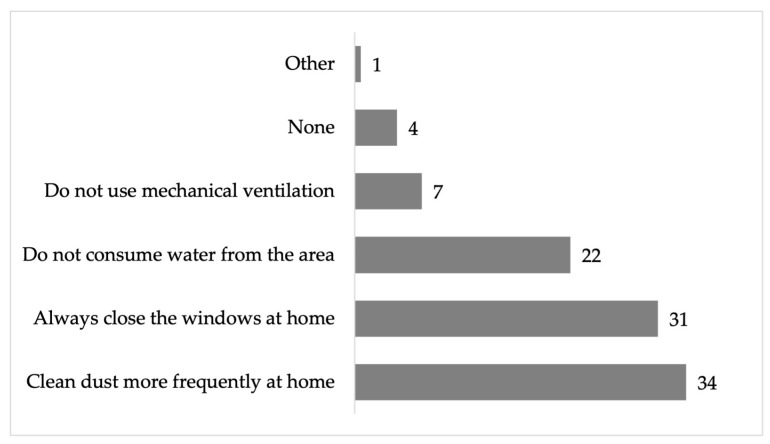
Percentage of actions taken to mitigate the impacts by inhabitants of the residential area surrounding the former Ávalos foundry.

Therefore, residents of the area indicated that they carry out strategies to mitigate the impacts, such as frequent cleaning of dust inside the homes (34%), keeping windows closed (31%), not consuming water from the area (22%), not using mechanical ventilation (7%), etc. Other responses mentioned included fumigation, watering the front of the homes, and applying lime around them (Figure 18).

**Figure 19:**
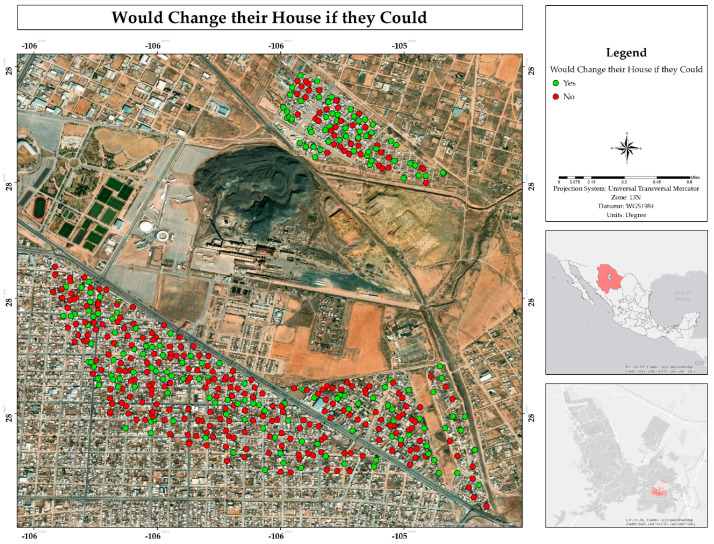
Spatial distribution of inhabitants that would change their house if given the opportunity from the residential area surrounding the former Ávalos foundry.

**Figure 20:**
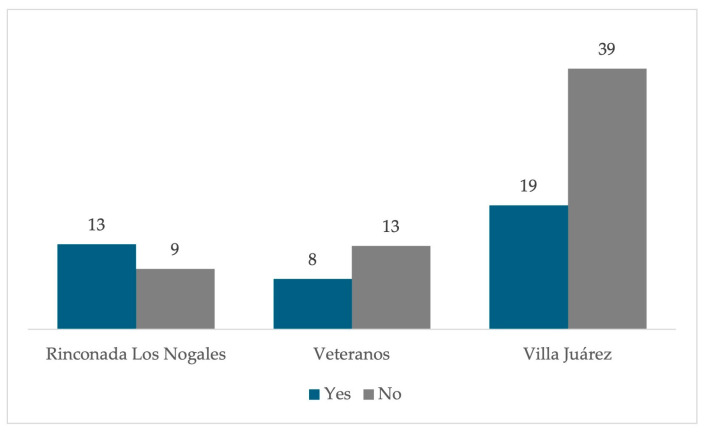
Percentage of residents in the residential area surrounding the former Ávalos foundry choosing to change their home if they had the opportunity, per neighborhood.

Even with all the above, 61% of respondents stated that they would not consider moving if the opportunity presented itself. In contrast, 39% indicated that they would be willing to make such a change (Figure 19). A greater positive response was observed in people who live in the Rinconada los Nogales neighborhood (Figure 20). These data reflect a majority tendency towards permanence in the current home, which could be influenced by factors such as owning the property and attachment to the place, even due to a lack of aspirations [13].

### 3.3. Expectations from Public Administration and Commitment

**Figure 21:**
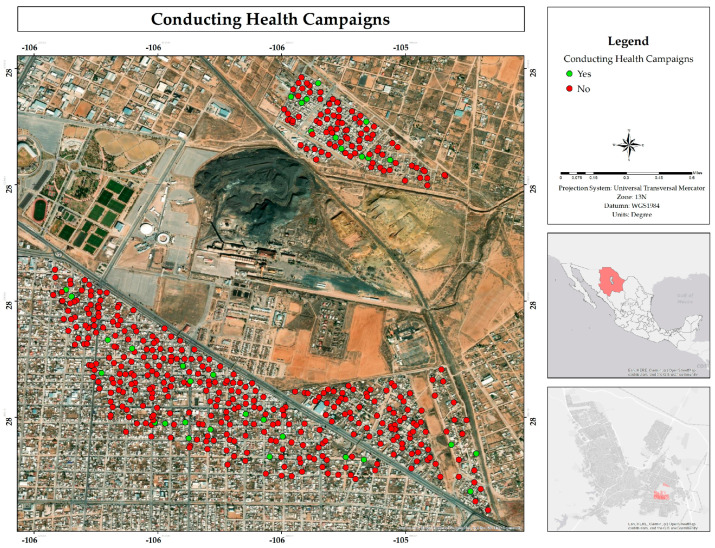
Spatial distribution of knowledge about health campaigns by public administration in the residential area surrounding the former Ávalos foundry.

Residents of the area indicated that they were not aware of health campaigns promoted by the public administration (93%) (Figure 21). This suggests a possible lack of dissemination of these initiatives or a limited role of the public administration in the sector, which could negatively impact the participation and scope of health promotion strategies.

**Figure 22:**
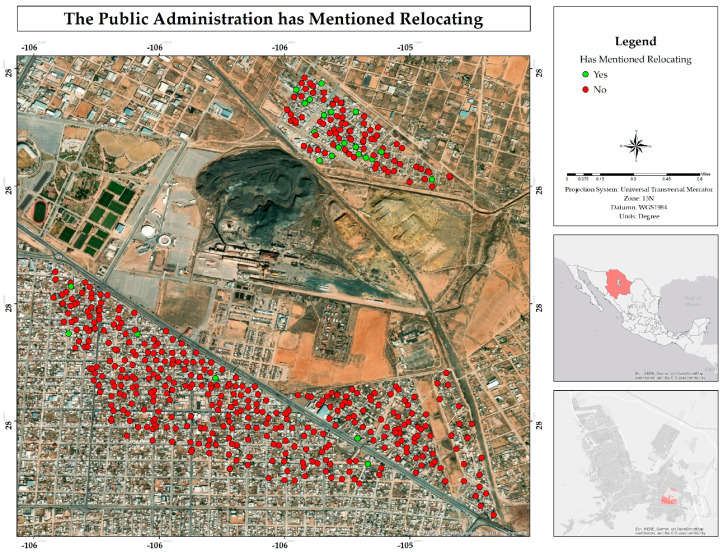
Spatial distribution of possible relocation by the public administration for residents of the residential area adjacent to the former Ávalos foundry.

**Figure 23:**
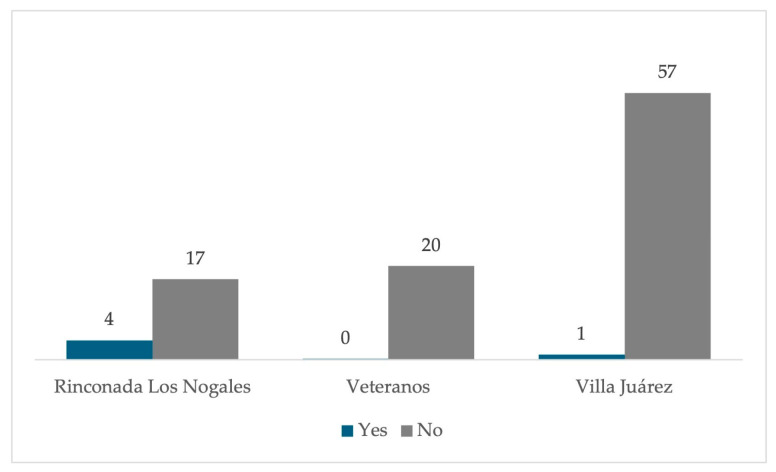
Percentage of possible relocation by the public administration for inhabitants of the residential area surrounding the former Ávalos foundry by neighborhood.

Residents mentioned that they were not aware of the implementation of relocation activities, while only 5% indicated that they were aware that these activities had been carried out (Figure 22), being more observed in the Rinconada Los Nogales than in the other two neighborhoods (Figure 23).

**Figure 24:**
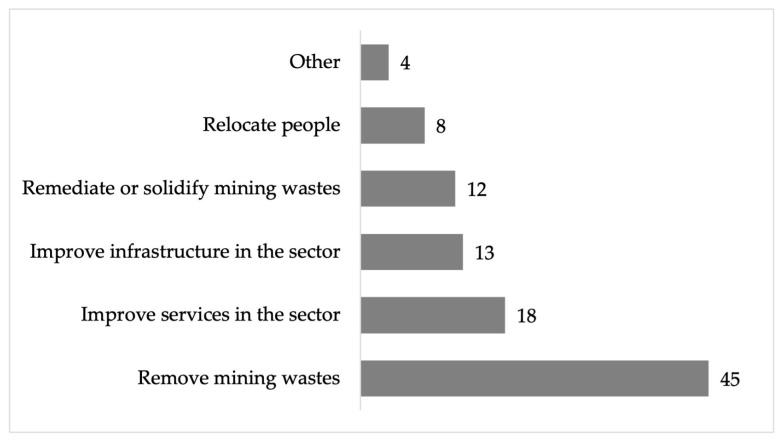
Percentage of requests to public administration from respondents in the residential area surrounding the former Ávalos foundry.

The residents of the area mentioned that they would ask to the public administration, to remove mining waste (45%), as well as to improve services (18%) and public infrastructure (13%) and to remediate the wastes (12%) or to relocate them (8%) (Figure 24).

In services, they were asked to improve the water supply, public lighting, and public transportation. In infrastructure, they pointed out the streets’ paving, painting pedestrian crossings, improving the local security, and increasing municipality cleaning.

Most of the population stated that they had no knowledge or had received no information on how to prevent exposure to mining waste (94%). Only 6% of respondents stated that they had received some type of training or information on prevention, especially in their children’s schools.

**Figure 25:**
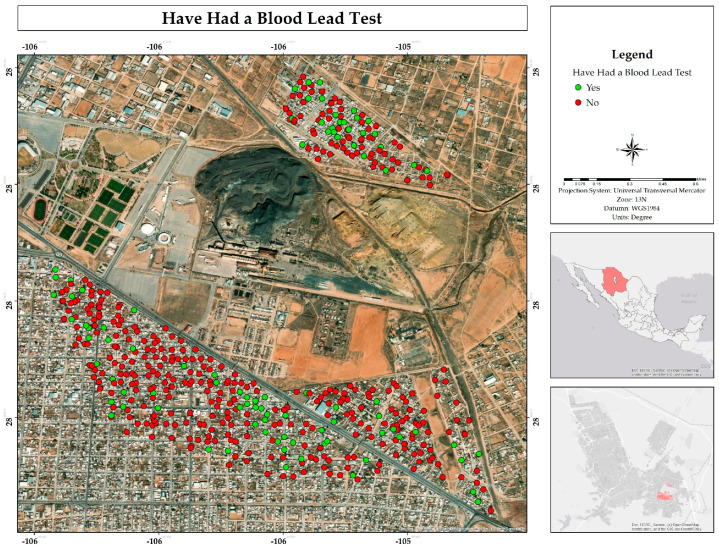
Spatial distribution of blood lead testing among respondents in the residential area surrounding the former Ávalos foundry.

**Figure 26:**
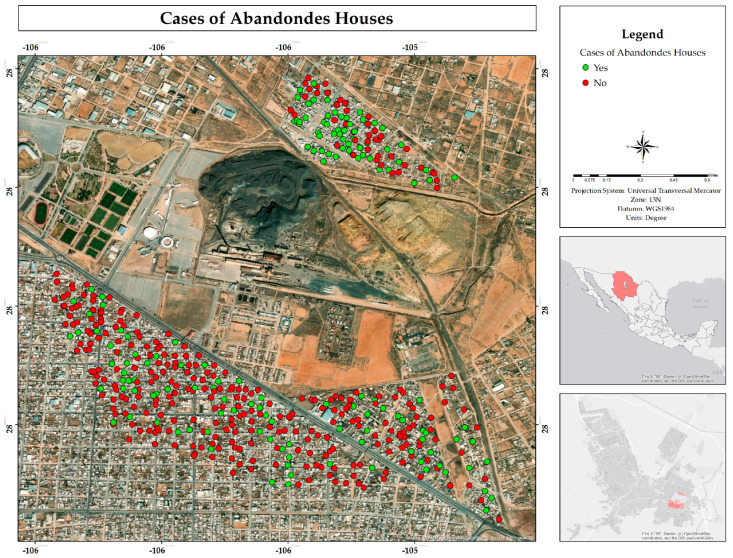
Spatial distribution of abandonment cases in the neighborhoods where respondents live in the residential area adjacent to the former Ávalos foundry.

In addition, the majority indicated that they had not undergone blood tests to determine the lead concentration in their blood; only 20% responded affirmatively (Figure 25). Furthermore, few were aware of actions by the authorities to mitigate contamination in the study area; only 7% indicated that such measures had been implemented. In addition to the above, 32% of respondents indicated that they knew a neighbor or person close to them who had abandoned their home due to exposure to mining waste (Figure 26).

**Figure 27:**
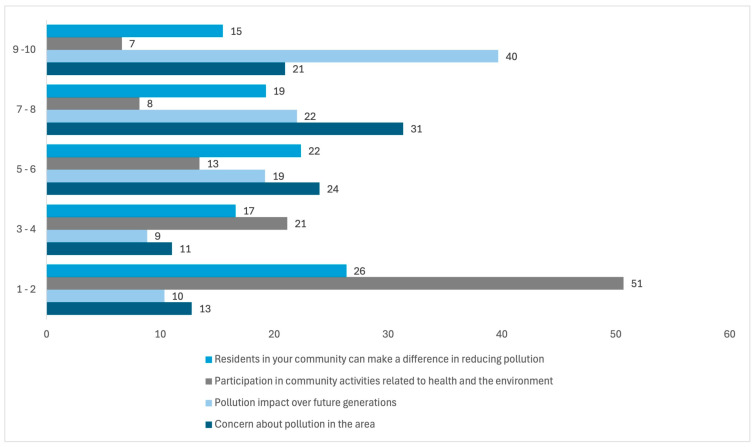
Percentages of concern about pollution, the impact over future generations, participation in community activities, and differences that the community can make to reduce pollution in the residential area surrounding the former Ávalos smelter.

Respondents indicated significant concern about local pollution (52%), with responses ranging from 7 to 10 (the range was from 1 less concern to 10 more concern). Likewise, they are concerned about the pollution impact on future generations (62%), with responses ranging from 7 to 10 (the range was from 1 less concern to 10 more concern). However, a low percentage (15%) of respondents expressed their willingness to participate in their community in activities related to health and the environment, with responses ranging from 7 to 10 (the range was from 1 less willingness to 10 more willingness). Only 34% of respondents answered affirmatively, rating the community’s ability to reduce pollution in their area with a score between 7 and 10 (Figure 27) (the range was from 1 less ability to 10 more ability).

### 3.4. Metals in Water

Several metals and metalloids regulated by NOM-127-SSA1-2021 were analyzed in tap water from residences in the study area: As, Cd, Cu, Cr, Fe, Mn, Ni, Pb, and Se [8]. Of these, Cd, Pb, and Se remained with values close to zero or not detected (ND). Other elements such as Be, Co, Ti, Tl, and Zn are not regulated by Mexican and/or international regulations, and most of these were determined as zero or not detected (ND) (Table 3).

**Figure 28:**
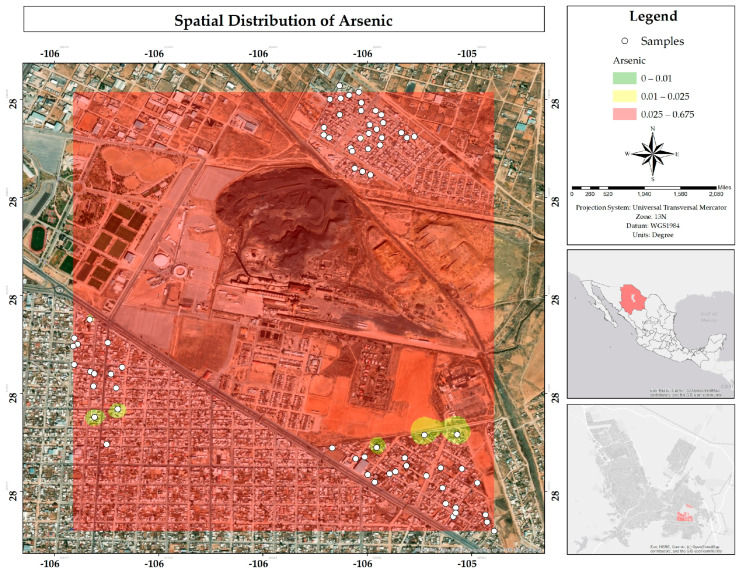
Spatial distribution of arsenic in mg/L in the water in the residential area of the former Ávalos smelter.

A total of 69% of the water samples exceeded the maximum permissible levels (MPLs) established by Mexican, American, and European regulations (0.01 mg/L in all) (Table 3). The map (Figure 28) shows in red and yellow all of the study area affected by this metalloid in the water. However, the elevated presence may be due to natural causes related to the geological characteristics of Chihuahua, as indicated by Ren et al. (2022), who reported that groundwater from wells around Chihuahua contains a high level of this metalloid; they also identified As minerals in the cavities of the Tertiary volcanic tuff in the northwestern part of the Tabalaopa basin, Chihuahua City [21].

**Figure 29:**
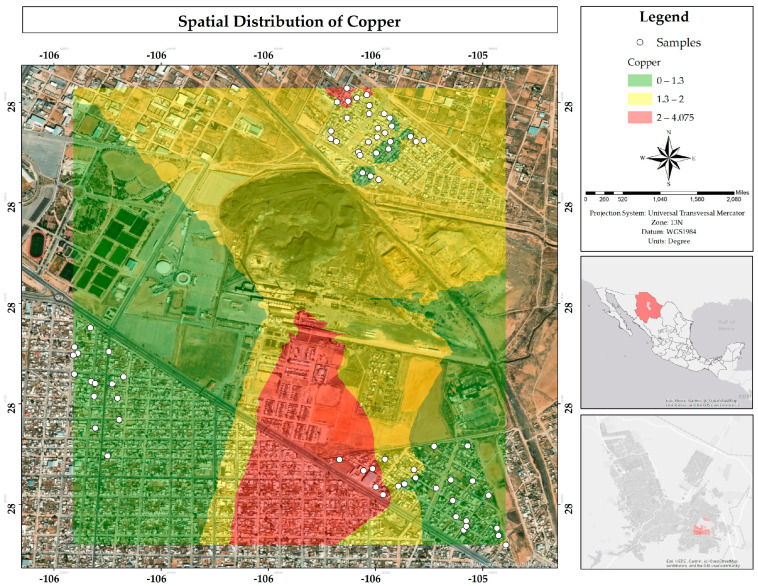
Spatial distribution of copper in mg/L in water in the residential area of the Ávalos smelter.

Most water samples (82%) fell within the MPLs for Cu established by Mexican and European regulations (2.0 mg/L in both), while 65% of the samples exceeded the MPLs defined in the United States (1.3 mg/L). The map (Figure 29) shows the Cu distribution above the MPLs in yellow and red in specific areas of the Veteranos and Rinconada Los Nogales neighborhoods. Gutiérrez et al. (2012) associated the elevated presence of Cu with its mobility from sediments originating in Tertiary volcanic rock, which predominates in the western region of the study area, an observation that is consistent with the proximity of the area analyzed in the present work [22].

**Figure 30:**
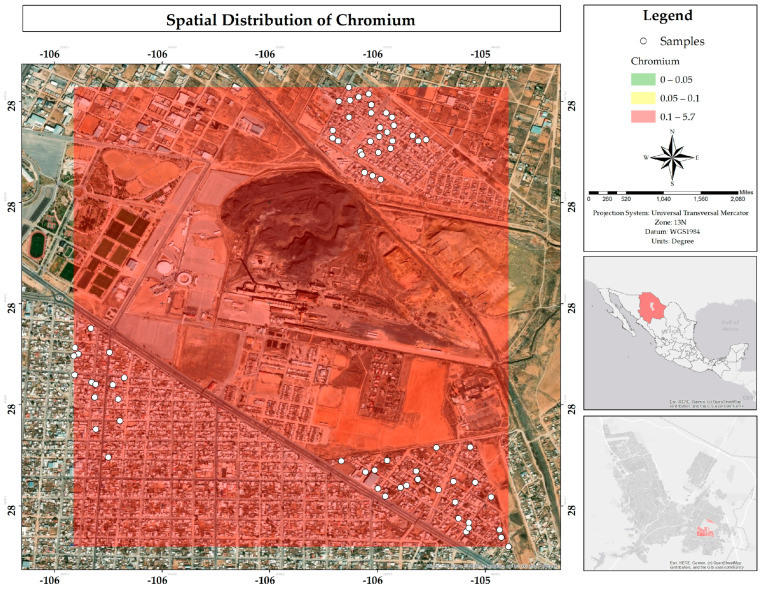
Spatial distribution of chromium in mg/L in water in the residential area of the Ávalos smelter.

A total of 99% of the samples exceed the MPLs established for Cr by Mexican (0.05 mg/L), American (0.10 mg/L), and European (0.025 mg/L) regulations (Table 3). The map shows in the color red that the entire study area is subject to the problem of this metal in the water (Figure 30). Cr in the aquatic environment mainly originates from natural sources; however, human activities have increased its concentration. It is also highly soluble in water and has prolonged persistence in the environment [23]. The biggest problem is seen in speciation, since CrVI is much more toxic [24]. Therefore, a new study evaluating the chemical form of chromium present in the drinking water is recommended to accurately determine the potential risk to public health.

**Figure 31:**
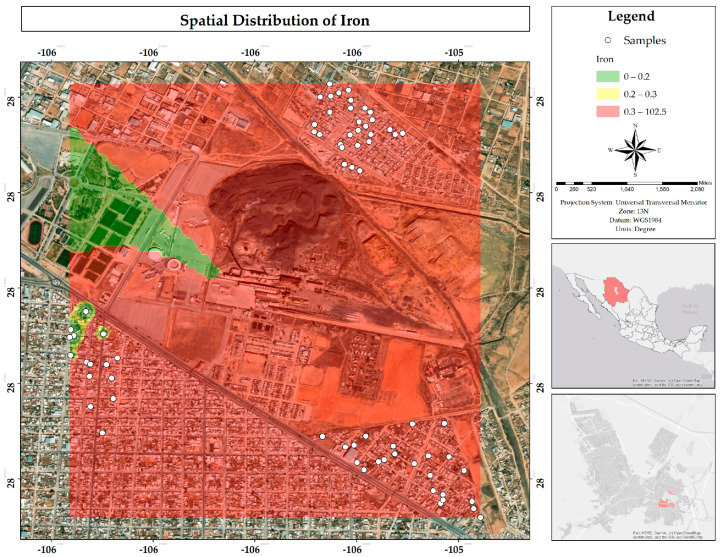
Spatial distribution of iron in mg/L in water in the residential area of the Ávalos foundry.

Most water samples (79%) exceed the MPLs for Fe established by Mexican (0.30 mg/L) and European (0.20 mg/L) regulations. The map (Figure 31) shows in yellow and red that almost the entire study area has high concentrations of this metal. The abundant presence of Fe has been documented in previous studies, such as that of Gutiérrez et al. (2021), who attribute its geogenic origin to two sources: primary and secondary. The primary origin refers to the dissolution of the parent rock, while the secondary origin is related to the release of contaminants retained in secondary minerals formed during the weathering of the original rock, including iron oxides and clays [25].

**Figure 32:**
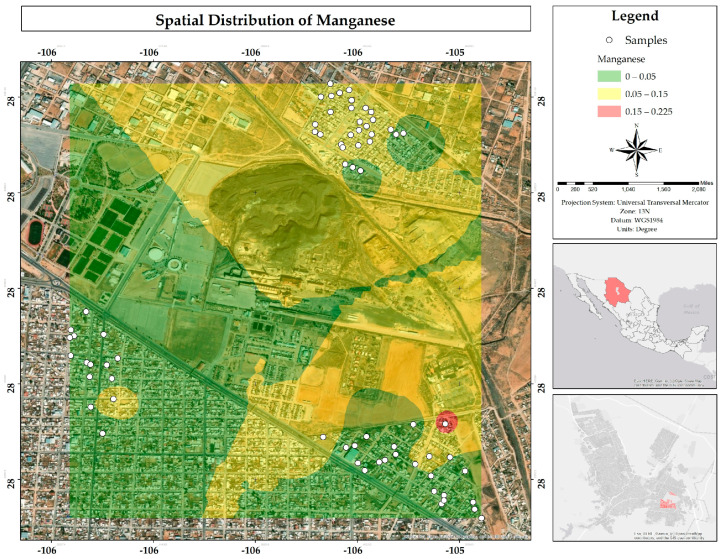
Spatial distribution of manganese in mg/L in water in the residential area of the Ávalos smelter.

A total of 97% of the water samples met the MPLs for Mn established by Mexican regulations (0.15 mg/L), while only 74% met the requirements of the European regulations (0.05 mg/L). The map (Figure 32) reveals that under Mexican regulations the problem is concentrated in very specific areas (in the color red), with largest area, in the color yellow, in the Rinconada Los Nogales neighborhood (North), where the European regulations were exceeded. The presence of this element in the state of Chihuahua has been documented by Ponce-González et al. (2024), who found significant concentrations in the southern area of Laguna de Encinillas, located in Chihuahua, Mexico. Its origin is geogenic and linked to the volcanic rocks of the surrounding mountain ranges. It is worth noting that the study area is located less than 140 km south of Laguna de Encinillas and coincides with the presence of these mountain ranges [26].

**Figure 33:**
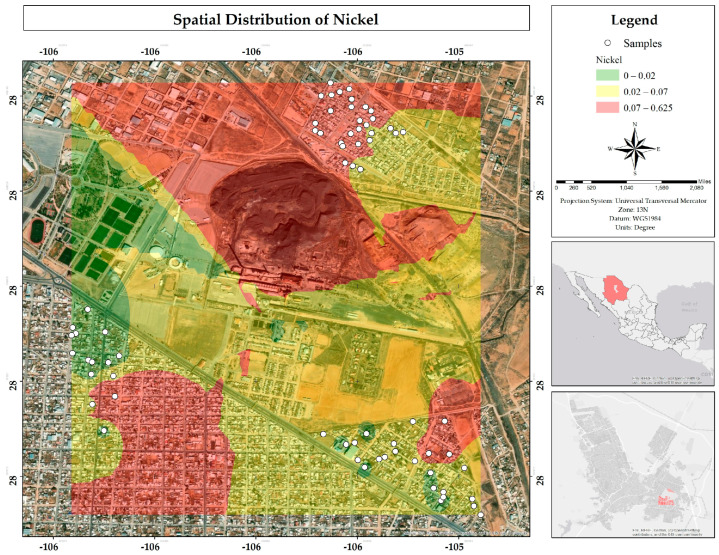
Spatial distribution of nickel in mg/L in water in the residential area of the Ávalos smelter.

A total of 66% of the samples met the MPL for Ni established by Mexican regulations (0.07 mg/L), while only 47% met the MPL stipulated by European regulations (0.02 mg/L). The map (Figure 33) shows that residential areas in the study area are affected by the presence of this metal in water in the colors yellow and red. The literature does not specifically report its presence, suggesting that its origin could be due to factors other than geology or the normal composition of the subsoil. However, it is important to highlight the high concentrations found in the core area of the study site, i.e., in the tailings.

## 4. Discussion

Most of the respondents were adults over 24 years of age, with a secondary or high school education level and a fixed income. The length of residence varied from 0 to more than 60 years, with those who have lived there between 0 and 20 years predominating. Housing choices were mainly based on affordability or inheritance. This resulted in a demographic composition that included both young families and older adults, many of whom have lived in the area for more than 20 years. Vulnerable groups such as children and pregnant women, are particularly affected, as affordable housing near environmental liabilities may significantly compromise their quality of life [27].

Despite the risks, most people feel comfortable in the area, which may be related to the sense of place attachment and the accessibility of essential services such as transportation routes, health centers, and schools, which favor their well-being [28]. However, this is contrasted by the majority who stated they would not have chosen to live there if they had known the risks, and, they have continued living in the area, revealing a tension and confusion between familiarity and fear, a phenomenon observed in other environmental justice contexts globally [29].

This contradiction reveals an important dynamic in socio-environmental perception: cognitive dissonance emerges when residents are simultaneously aware of risk and dependent on the place they live. Similar patterns have been observed in global case studies in Argentina, India, and the United States, where historically marginalized communities often normalize contamination due to lack of alternatives or social support [30,31]. In such cases, perception is not merely a function of information access, but also of emotional, economic, and cultural ties to the land [32].

Furthermore, the lack of effective communication regarding environmental hazards may be a factor in the passive response of the residents to the threat. Environmental risks are often underplayed in communities where vulnerability is intertwined with socio-economic struggles, and this mitigates the willingness to act upon the perceived threat [33]. Studies show that people with lower socioeconomic status are often less aware of the potential dangers of environmental hazards because their focus is more on their immediate survival needs [34].

Residents consider the water from tanks or the public network to be of poor quality, which leads them to use filters, buy bottled water, or obtain water from vendors due to the uncertainty about the effects of contamination [29]. Despite the adoption of personal protective measures, the proximity to toxic residues continues to pose long-term health risks.

Although the risk of developing diseases increases with the proximity to contaminated sites [35], many residents reported not having symptoms. Respiratory diseases associated with contamination, such as allergies and asthma, were the most reported health issues [36]. Indirect questions revealed cases of cancer, anemia, kidney problems, and birth defects, such as Down syndrome and autism. These findings echo global trends in areas exposed to industrial pollution, including Flint, Michigan, Puyango river, Ecuador and La Oroya, Peru, where long-term exposure to heavy metals correlates with developmental delays and chronic illness in children [33,37,38].

Dust was identified as the main environmental problem, affecting homes, water storage, and daily activities. Dust sources may include the mining wastes from the former foundry (tailings and ground slag) and unpaved streets (approximately 30% [6]). While residents mitigate the impact through personal practices like cleaning and closing windows, this does not reduce overall exposure. Regarding the place attachment, most residents of older neighborhoods such as Villa Juárez and Veteranos would not want to move, unlike the Rinconada Los Nogales neighborhood, where the feeling of belonging is lower [39].

Environmental impacts may not be evident in the short term, and their assessment is complicated by the invisibility in the relationship between pollution and health [40]. In these conditions, the residents often minimize the importance of addressing the environmental liability, associating their illnesses with age or work-related factors, rather than environmental causes [41], which diminishes the perceived need for remediation [42]. Furthermore, in highly polluted areas, the affected population tends to attribute responsibility with the government or higher entities [29]. To improve the perception of government action, the needs of affected residents must be effectively addressed, in line with recommendation No. 91/2019 of the CNDH [6].

The environmental liability in the area affects not only the houses, but also nearby places such as the sports complex, a medical unit, and the market, exposing those who visit them to contamination [43]. Regarding health, most residents are unaware of any prevention campaigns being conducted, and in the Rinconada Los Nogales neighborhood, some residents have been relocated by the State Government [6]. Likewise, a majority believes that no remediation measures have been implemented in the area, although minor works have been carried out. However, the source of the problem has not been addressed: the mining tailings and contaminated soil from the former smelter. Additionally, there are reports of home abandonment due to pollution, especially in Rinconada Los Nogales, which is the most affected neighborhood due to its proximity to mining tailings (Figure 1).

Despite concern about future generations, few residents expressed willingness to participate in environmental or health initiatives. This apathy may stem from resignation or a perceived lack of efficacy, a sentiment observed in polluted communities worldwide [30]. Nevertheless, a substantial portion of respondents still believe collective action could make a difference, indicating a latent potential for community engagement if supported by institutions. This suggests that although residents are aware of the magnitude of the problem, they prefer to ignore it rather than act [44].

Although several metals have very low or zero concentrations in the tap water, arsenic, copper, chromium, iron, manganese, and nickel exceed the maximum permissible limits for human consumption. Regardless of whether the source is natural or anthropogenic due to mining waste, the population is exposed to these contaminants, which can harm their health. They can cause serious health problems, including skin, bladder, and lung cancer, in addition to affecting cardiovascular and neurological function [45]. Cu is an essential nutrient for humans, animals, and plants, but it can pose risks to human health with elevated exposure, including gastrointestinal problems [46]. Cr is one of the main elements required for the metabolism of carbohydrates and fats. Further, Cr increases insulin sensitivity by correspondingly increasing the binding of insulin to cells and the number of insulin receptors. The biggest problem is seen in speciation, since CrVI is much more toxic [24]. Iron is an essential mineral required for various bodily functions, but its overload causes severe health problems in humans, such as liver cancer, diabetes, cirrhosis, heart diseases, and infertility [47]. Although, Mn is essential at low concentrations, high levels can cause neurological problems, especially in children, and it affects water quality [48]. Ni can cause adverse effects on the respiratory and renal systems [49].

González-Horta et al. (2015) reported that a significant number of residents in the southwestern part of the city of Chihuahua (this area includes Ávalos), are chronically exposed to high As and fluoride (F) levels in drinking water. They also report that antagonistic and synergistic effects have been documented between these two elements and their consequences, suggesting the need to take immediate measures to reduce exposure [50].

The global relevance of this study lies in its contribution to understanding how historically marginalized urban populations experience, perceive, and respond to environmental contamination. Worldwide, more than 200 million people live near contaminated sites, many of which stem from mining or heavy industry [51]. These communities often lack the resources to relocate or demand remediation. By documenting the perceptions, adaptations, and contradictions of the residents in Ávalos, this research provides insight that can inform risk communication, environmental health policy, and remediation planning not only in Mexico but also in similarly affected regions in Latin America, Asia, and Sub-Saharan Africa [52,53]. Such studies are critical to advancing global environmental justice efforts and achieving Sustainable Development Goals related to health, clean water, and sustainable cities [54].

## 5. Conclusions

The population surrounding the mining tailings of the former Ávalos smelter suffers economic effects, such as having to purchase bottled water, rely on retail water sources, or install filters, and in some cases, abandon their homes due to environmental exposure. Socially, residents report increased cleaning routines, keeping doors closed, and avoiding mechanical ventilation due to excessive dust, alongside a persistent concern about the problem in the area. There are also environmental effects; there is widespread exposure through water, soil, and air to contaminants originating from mining wastes. Additionally, public health is affected by some illnesses, particularly respiratory and dermatological conditions.

Although many inhabitants consider their quality of life as comfortable, several stated they would not have chosen to live in the area had they been aware of the associated risks. In Rinconada Los Nogales, the majority would be willing to relocate, where in Veteranos and Villa Juárez, strong emotional or hereditary ties to housing deter relocation. The perception of the situation remains mixed: while some demand urgent action, most feel neglected by the authorities. Nevertheless, the historical significance of the former smelter offers potential for revitalized space that could bring a positive impact to the area and the community.

The metal concentration in water reveals significant variations in the quality of the tap water. The elevated presence of arsenic, copper, iron, and manganese poses serious public health challenges, whether originating from natural sources or anthropogenic mining waste. The findings of chromium and nickel further underscore the need for updated regulatory criteria and strengthened monitoring and mitigation strategies. These results highlight the importance of additional studies focused on contaminant speciation and exposure pathways to guide more effective interventions.

Despite the relevance of the findings, the study presents several limitations that should be considered. The reliance on self-reported health and perception data introduces subjectivity and possible bias, potentially affecting the accuracy of conclusions about health conditions. The absence of biomonitoring or comprehensive environmental sampling, especially in soil and air, limits the ability to confirm exposure pathways or establish causal links. Moreover, without a control group or comparative analysis, it is difficult to isolate the effects of mining waste from other environmental or social determinants. Finally, the study’s cross-sectional nature offers only a snapshot in time, without capturing temporal dynamics or long-term trends.

Even with these limitations, this study contributes valuable insights into how socio-environmental perception, health, and historical industrial activity intersect in vulnerable urban contexts. At a global scale, it reinforces the need to address urban environmental liabilities and their overlooked health impact.

This study suggests immediate interventions, such as public awareness campaigns, targeted health monitoring, and a reduction in residential density near contaminated zones. Housing development should be restricted in already consolidated areas, and environmental remediation efforts should be accelerated. Cross-sector collaboration between public authorities, educational institutions, and civil society is essential to mobilize funding and implement sustainable solutions to prioritize environmental justice and public health.

## Figures and Tables

**Table 1:** Housing in the study area (Source: INEGI, 2020).

Total number of housing units	5773
Total number of private housing units	5627
Occupied private housing units	4634
Unoccupied private housing units	853

**Table 2:** Population distribution by age cohorts in the study area (Source: INEGI, 2020).

Total population	14,187
Female population	7198
Male population	6868
Population aged 0 to 14 years	2935
Population aged 15 to 29 years	3238
Population aged 30 to 59 years	5533
Population aged 60 years and older	2190
Population with disabilities	513

**Table 3:** Average concentrations of metals and metalloids in the water compared to the maximum permissible levels defined in Mexican, American, and European regulations.

Metal	Mexico NOM-127-SSA1-2021 (mg/L)	United States Safe Drinking Water Act (EPA) (mg/L)	Directive (EU) 2020/2184 of the European Parliament and of the Council 2020/2184 (mg/L)	Mean Concentration (mg/L) n = 68	Standard Deviation
Arsenic	0.010	0.010	0.010	0.072	0.093
Cadmium	0.003	0.005	0.005	0.004	0.030
Copper	2.000	1.300	2.000	1.356	0.705
Chromium	0.050	0.100	0.025	2.167	0.820
Iron	0.300	Not regulated	0.200	7.102	13.236
Manganese	0.150	Not regulated	0.050	0.050	0.046
Nickel	0.070	Not regulated	0.020	0.078	0.126
Lead	0.010	0.010	0.005	0.001	0.006
Selenium	0.040	0.050	0.020	0.005	0.026
Beryllium	Not regulated	0.004	Not regulated	0.001	0.004
Thallium	Not regulated	0.002	Not regulated	0.000	0.000
Cobalt	Not regulated	Not regulated	Not regulated	0.000	0.000
Titanium	Not regulated	Not regulated	Not regulated	0.001	0.004
Zinc	Not regulated	Not regulated	Not regulated	0.003	0.015

## Data Availability

The data presented in this study are available on request from the corresponding author, due to the sensitivity of the information and that it may generate speculation is not public information.

## Abbreviations

The following abbreviations are used in this manuscript:

CNDH	National Human Rights Commission
PDU	Urban Development Plan of the Municipality of Chihuahua 2040
ICP-OES	Inductively Coupled Plasma Optical Emission Spectrometer
EU	European Union
IDW	Inverse Distance Weighted
MPL	Maximum Permissible Level
